# ﻿Complete mitochondrial genomes of *Boigakraepelini* and *Hebiuscraspedogaster* (Reptilia, Squamata, Colubridae) and their phylogenetic implications

**DOI:** 10.3897/zookeys.1124.87861

**Published:** 2022-10-17

**Authors:** Shuangshuang Shan, Yu Wang

**Affiliations:** 1 College of Chemistry and Life Sciences, Zhejiang Normal University, Jinhua, China Zhejiang Normal University Jinhua China; 2 Key Laboratory of Wildlife Biotechnology, Conservation and Utilization of Zhejiang Province, Zhejiang Normal University, Jinhua, China Zhejiang Normal University Jinhua China

**Keywords:** Colubrinae, mitogenomes, Natricinae, phylogenetic analysis, protein-coding genes

## Abstract

The complete sequence of the mitochondrial genome is a powerful tool for studying phylogenetic relationships and molecular evolution in various species. In this work, the mitogenomes of *Boigakraepelini* and *Hebiuscraspedogaster* were sequenced and characterized for the first time. The lengths of the *B.kraepelini* and *H.craspedogaster* mitogenomes were 17,124 bp and 17,120 bp, respectively, and both included 13 protein-coding genes, 22 tRNAs, two rRNAs and two control regions. The arrangements of these mitochondrial genes were the same in *B.kraepelini* and *H.craspedogaster*. In addition, both genome compositions showed A+T bias (59.03%, 60.93%) and had positive AT skews (0.179, 0.117) and negative GC skews (-0.397, -0.348). The phylogenetic results illustrated a close relationship between *B.kraepelini* and the genus *Lycodon*. Moreover, *H.craspedogaster* was clustered with other *Hebius* snakes and closely related to other Natricinae species. These results will provide references for further research on the phylogeny of Colubridae.

## ﻿Introduction

Colubridae is a family with high species diversity in the suborder Serpentes, which is distributed on almost all continents (Pough et al. 2004). The hierarchical classification of Colubridae can be divided into eight subfamilies (Ahaetuliinae, Calimariinae, Colubrinae, Dipsadinae, Grayiinae, Natricinae, Pseudoxenodontinae, and Sibynophiinae) based on molecular markers and morphological characters (Figueroa et al. 2016; Zaher et al. 2019). However, the relationships among these subfamilies and the relationships among genera in a specific subfamily are still unclear since varied genes have been applied in phylogenetic statistics (Lawson et al. 2005; Pyron et al. 2013a, b; Figueroa et al. 2016; Zheng and Wiens 2016; Zaher et al. 2019). *Boigakraepelini* Stejneger, 1902 and other *Boiga* species are arboreal snakes distributed in Asia, Australia and Pacific islands (Weinell et al. 2021). As a genus belonging to Colubridae, *Boiga* species share the characteristics of rapid movement with other colubrid species, with the exception of posterior groove teeth and low toxicity. Species listed in the genus *Hebius* are mainly distributed in the eastern, southern and southeastern regions of Asia (Guo et al. 2012). They are usually small- to medium-sized snakes and considered innocuous (Zhao 2006). More evidence should be obtained to understand their phylogenetic position since *Hebius* is a relatively new genus split from the genus *Amphiesma* in recent years (Guo et al. 2014).

The mitochondrial genomes of snakes are circular molecules that contain 13 protein-coding genes (PCGs), 22 transfer RNA (tRNA) genes, 2 ribosomal RNA (rRNA) genes, and one or two duplicate control regions. Due to the advantages of small size, matrilineal inheritance, relatively stable genetic structure, easy amplification and high evolutionary rate, partial or full sequences of the mitogenome have been extensively used in molecular evolution, comparative and evolutionary genomics, phylogenetics and population genetics research in various animal species (Kim et al. 2018; Huang et al. 2019). With the development of sequencing technology, a large number of animal mitochondrial genomes have been sequenced and sequences are becoming more accessible (Zhou et al. 2016; Wang et al. 2019). As an informative molecular marker, phylogenetic relationships based on the mitogenome often result in better resolution, reliability and robustness than those of other molecular markers (Madsen et al. 2001). A previous study showed that *B.kraepelini* was the sister lineage to all 23 other *Boiga* species (Weinell et al. 2021) and that *Hebius* is a monophyletic genus (Guo et al. 2014) based on a few gene fragments. Here, the complete mitogenomes of *B.kraepelini* and *H.craspedogaster* Boulenger, 1899 were sequenced, annotated and characterized for the first time. To better understand the relationships among Colubridae, complete sequences of 13 mitochondrial PCGs from 38 species of Colubridae and two outgroup species were used to construct a comprehensive phylogenetic tree.

## ﻿Materials and methods

### ﻿Sampling and DNA extraction

Specimens of *B.kraepelini* and *H.craspedogaster* were collected from Jinhua, China (29°12'N, 119°37'E). Total genomic DNA (gDNA) was extracted from tail muscle using a Rapid Animal Genomic DNA Isolation Kit (Sangon Biotech, China) according to the manufacturer’s instructions.

### ﻿PCR amplification and sequencing

Conventional polymerase chain reaction (PCR) assays were conducted to amplify the complete mitogenomes of *B.kraepelini* and *H.craspedogaster*. The specific primers were designed based on the known nucleotide sequences (Suppl. material 1: Table S1) (Guo et al. 2012; Li et al. 2020; Weinell et al. 2021). Amplification was performed in a total volume of 50 μL, which contained 25 μL of 2× Es Taq MasterMix (CWBIO, China) of 3.0 mM MgCl_2_, each dNTP at 0.40 mM and 1.0 U of Taq DNA polymerase per μL, 2 μL each of forward and reverse primers (10 μM), 2 μL template DNA and 19 μL of sterilized water. The thermal cycling procedure was applied as follows: an initial pre-denaturation step at 95 °C for 3 min, followed by 35 cycles at 95 °C for 30 s, 60 °C for 45 s, and 72 °C elongation for 1–4 min (depending on the size of fragments), with a final extension at 72 °C for 10 min. The PCR products were recycled and purified using 1.5% agarose gel electrophoresis and genotyped using Sanger sequencing by Sangon Biotech (Shanghai) Co., Ltd., China.

### ﻿Sequence assembly and gene annotation

The obtained sequences were identified using the Basic Local Alignment Search Tool (BLAST) from NCBI and were assembled using SeqMan software (DNAStar Inc., USA). The complete mitochondrial sequences were annotated by the MITOS web server (http://mitos.bioinf.uni-leipzig.de/index.py) (Bernt et al. 2013) and corrected manually. Transfer RNA (tRNA) genes were identified and predicted in the tRNAscan-SE search server (http://lowelab.ucsc.edu/tRNAscan-SE/) (Lowe and Chan 2016) using the vertebrate genetic code, and their secondary structures were visualized in the Forna web server (http://rna.tbi.univie.ac.at/forna/forna.html) (Kerpedjiev et al. 2015). The base composition of the mitogenome and the relative synonymous codon usage (RSCU) of PCGs were determined using MEGA X (Kumar et al. 2018). The skewness of nucleotide composition was measured according to the following formulas: AT-skew = [A – T] / [A + T] and GC-skew = [G – C] / [G + C] (Perra and Kocher 1995). Graphical maps of the complete mitochondrial genomes were drawn using the online visualization tool mtviz (http://pacosy.informatik.uni-leipzig.de/mtviz).

### ﻿Phylogenetic analyses

To understand the phylogenetic positions of *B.kraepelini* and *H.craspedogaster*, the complete mitochondrial sequences of 13 PCGs in 38 previously available species of Colubridae and two outgroups (*Najaatra* and *Hypsiscopusplumbea*) were obtained from GenBank (Table 1). Since nucleotide sequences with substitution saturation has previously plagued phylogenetic analyses, the suitability for phylogenetic tree construction from the dataset was tested first using DAMBE7 software (Xia 2018). The nucleotide sequences were aligned through the MAFFT v.7.475 program with default settings (Katoh et al. 2002). Sequence gaps and poorly aligned regions were removed using Gblocks v.0.91 (Castresana 2000). The best-fit substitution model for the dataset was GTR + I + G by jModelTest v.2.1.10 (Darriba et al. 2012) based on Akaike Information Criterion (AIC). Phylogenetic analyses were performed using Bayesian inference (BI) and maximum likelihood (ML) methods by MrBayes v.3.2.7 (Ronquist and Huelsenbeck 2003) and IQ-TREE v.2.1.2 (Minh et al. 2020), respectively. Four independent runs were conducted using the default settings for 5,000,000 generations with a sampling frequency of 1000 and a burn-in of 25% of samples with Bayesian analyses. Only when the average standard deviation of the split frequencies was less than 0.01 and the effective sampling size greater than 200 were the Markov chain Monte Carlo (MCMC) chains considered convergent. All parameters were assessed by Tracer v.1.7.1 (Rambaut et al. 2018). In the ML analyses, branch support was estimated by 1000 ultrafast bootstrap replicates. The resultant trees were visualized using FigTree v.1.4.4 (http://tree.bio.ed.ac.uk/software/figtree/).

**Table 1.** Mitochondrial genome sequences with GenBank accession numbers used in this study.

Family	Species	Accession No.
Colubridae	*Boigakraepelini*	This study
	*Elapheanomala*	KP900218
	*Elaphebimaculata*	KM065513
	*Elaphedione*	MH460961
	*Elaphecarinata*	KU180459
	*Elaphedavidi*	KM401547
	*Elapheporyphyracea*	GQ181130
	*Elaphequadrivirgata*	AB738958
	*Elaphequatuorlineata*	MK334307
	*Elaphesauromates*	MK070315
	*Elapheschrenckii*	KP888955
	*Elaphetaeniurus*	KC990021
	*Euprepiophisperlacea*	KF750656
	*Gonyosomafrenatum*	MW413812
	*Lycodonflavozonatus*	KR911720
	*Lycodonrufozonatum*	KF148622
	*Lycodonruhstrati*	MK867843
	*Lycodonsemicarinatus*	AB008539
	*Oligodonchinensis*	MK347418
	*Oocatochusrufodorsatus*	KC990020
	*Orientocoluberspinalis*	MT304473
	*Pantherophisslowinskii*	DQ523162
	*Pituophiscatenifersayi*	KU833245
	*Ptyasdhumnades*	KF148621
	*Ptyasmajor*	KF148620
	*Ptyasmucosa*	KT982276
	*Thermophisbaileyi*	MF326642
	*Thermophisshangrila*	MF066951
	*Thermophiszhaoermii*	GQ166168
	*Hebiuscraspedogaster*	This study
	*Hebiusoptatum*	MN427890
	*Hebiusvibakariruthveni*	KP684155
	*Nerodiasipedon*	JF964960
	*Opisthotropisguangxiensis*	MT571495
	*Opisthotropislatouchii*	MK570292
	*Pseudagkistrodonrudis*	MW327508
	*Rhabdophistigrinus*	KU641019
	*Pseudoxenodonstejnegeri*	MW018358
	*Sibynophischinensis*	KF360246
	*Sibynophiscollaris*	JN211315
Elapidae	*Najaatra*	EU913475
Homalopsidae	*Hypsiscopusplumbea*	DQ343650

## ﻿Results and discussion

### ﻿Genome content and organization

**Figure 1.**
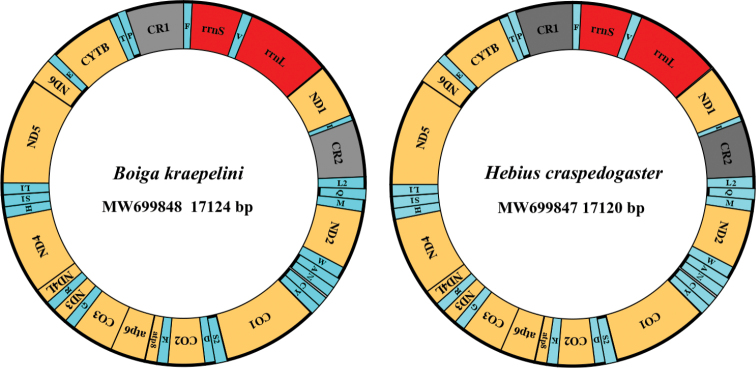
Graphical maps of *Boigakraepelini* and *Hebiuscraspedogaster* mitogenomes. Thirteen protein-coding genes (PCGs) and two ribosomal RNA genes (rrnS and rrnL) are shown with standard abbreviation. Twenty-two transfer RNA (tRNA) are abbreviated by a single letter. CR1 and CR2 are two putative control regions.

The complete mitogenomes of *B.kraepelini* and *H.craspedogaster* (GenBank accession numbers: MW699848 and MW699847, respectively) were closed double stranded DNA molecules 17,124 bp and 17,120 bp in length, respectively (Fig. 1). Both contained 37 typical mitochondrial genes, including 13 PCGs, 22 tRNA genes, two rRNA genes (*rrnS* and *rrnL*), two putative control regions (*CRs*) and one origin of light-strand replication (*O_L_*). Among these genes, 28 were encoded on the heavy strand, while the remaining nine genes, including one PCG (*nad6*) and eight tRNAs (*trnQ*, *trnA*, *trnN*, *trnC*, *trnY*, *trnS2*, *trnE* and *trnP*), were located on the light strand (Fig. 1, Table 2). The arrangement of genes in these two species was consistent with other species of snakes (Dong and Kumazawa 2005; Li 2014; Qian 2018). The nucleotide composition of *B.kraepelini* was 34.81% A, 24.22% T, 28.61% C and 12.36% G, and that of *H.craspedogaster* was 34.04% A, 26.89% T, 26.34% C and 12.73% G. Both species showed a significant bias toward A + T (59.03% for *B.kraepelini* and 60.93% for *H.craspedogaster*). In addition, the positive AT skew (0.179 and 0.117) and negative GC skew (-0.397 and -0.348) for *B.kraepelini* and *H.craspedogaster*, respectively, indicated higher frequencies of A and C than of T and G present in the whole mitogenome (Table 3). The biased A+T content and skewness in nucleotide composition of *B.kraepelini* and *H.craspedogaster* were highly similar to those of other Colubridae species (He et al. 2010; Sun et al. 2017; Wang et al. 2019).

**Table 2.** Summary of the mitogenomes of *Boigakraepelini* and *Hebiuscraspedogaster*.

Gene	Strand	*Boigakraepelini*			*Hebiuscraspedogaster*			Anti-codon
		Location	Size (bp)	Start / Stop codon	Location	Size (bp)	Start / Stop codon	
*trnF*	H	1–61	61	–	1–63	63	–	GAA
*rrnS*	H	62–978	917	–	64–988	925	–	–
*trnV*	H	979–1042	64	–	989–1052	64	–	TAC
*rrnL*	H	1043–2498	1456	–	1053–2497	1445	–	–
*nad1*	H	2515–3478	964	ATA/T	2519–3482	964	ATA/T	–
*trnI*	H	3479–3544	66	–	3483–3546	64	–	GAT
*CR2*	–	3545–4556	1012	–	3547–4537	991	–	–
*trnL2*	H	4557–4629	73	–	4538–4610	73	–	TAA
*trnQ*	L	4631–4701	71	–	4611–4681	71	–	TTG
*trnM*	H	4703–4764	62	–	4682–4744	63	–	CAT
*nad2*	H	4765–5794	1030	ATT/T	4745–5771	1027	ATG/T	–
*trnW*	H	5795–5859	65	–	5772–5838	67	–	TCA
*trnA*	L	5860–5922	63	–	5841–5905	65	–	TGC
*trnN*	L	5923–5994	72	–	5906–5978	73	–	GTT
*O_L_*	–	5997–6031	35	–	5981–6015	35	–	–
*trnC*	L	6030–6089	60	–	6014–6072	59	–	GCA
*trnY*	L	6090–6151	62	–	6074–6135	62	–	GTA
*cox1*	H	6144–7754	1611	ATA/AGG	6128–7738	1611	ATA/AGG	–
*trnS2*	L	7745–7811	67	–	7729–7795	67	–	TGA
*trnD*	H	7812–7875	64	–	7796–7860	65	–	GTC
*cox2*	H	7876–8560	685	ATG/T	7862–8546	685	ATG/T	–
*trnK*	H	8561–8624	64	–	8547–8609	63	–	TTT
*atp8*	H	8626–8784	159	ATG/TAA	8610–8774	165	ATG/TAA	–
*atp6*	H	8775–9455	681	ATG/TAA	8765–9445	681	ATG/TAA	–
*cox3*	H	9455–10238	784	ATG/T	9445–10228	784	ATG/T	–
*trnG*	H	10239–10299	61	–	10229–10289	61	–	TCC
*nad3*	H	10300–10642	343	ATT/T	10290–10632	343	ATA/T	–
*trnR*	H	10643–10707	65	–	10633–10696	64	–	TCG
*nad4L*	H	10708–10998	291	ATG/TAA	10697–10987	291	ATG/TAA	–
*nad4*	H	10998–12335	1338	ATG/TAA	10987–12321	1335	ATG/TAA	–
*trnH*	H	12336–12400	65	–	12322–12386	65	–	GTG
*trnS1*	H	12402–12458	57	–	12388–12444	57	–	GCT
*trnL1*	H	12456–12526	71	–	12442–12512	71	–	TAG
*nad5*	H	12527–14290	1764	ATG/TAA	12514–14295	1782	ATG/TAA	–
*nad6*	L	14286–14798	513	ATG/AGG	14291–14809	519	ATG/AGA	–
*trnE*	L	14799–14860	62	–	14810–14872	63	–	TTC
*cob*	H	14861–15977	1117	ATG/T	14873–15989	1117	ATG/T	–
*trnT*	H	15978–16043	66	–	15990–16053	64	–	TGT
*trnP*	L	16044–16105	62	–	16054–16115	62	–	TGG
*CR1*	–	16106–17124	1019	–	16116–17120	1005	–	–

**Table 3.** Nucleotide composition of *Boigakraepelini* and *Hebiuscraspedogaster* mitogenomes; the values for *B.kraepelini* are shown before the slash (/) and of *H.craspedogaster* are listed after the slash.

	A %	T %	G %	C %	A+T %	AT-skew	GC-skew
Mitogenome	34.81 / 34.04	24.22 / 26.89	12.36 / 12.73	28.61 / 26.34	59.03 / 60.93	0.18 / 0.12	-0.40 / -0.35
PCGs	35.48 / 34.72	23.53 / 27.19	11.00 / 11.15	29.99 / 26.94	59.01 / 61.92	0.20 / 0.12	-0.46 / -0.42
tRNAs	33.38 / 32.82	24.39 / 25.32	16.80 / 17.60	25.44 / 24.26	57.77 / 58.13	0.16 / 0.13	-0.21 / -0.16
*rrn*S	36.75 / 36.97	18.54 / 20.11	17.78 / 17.84	26.94 / 25.08	55.29 / 57.08	0.33 / 0.30	-0.21 / -0.17
*rrnL*	40.80 / 39.65	20.33 / 21.25	15.38 / 16.40	23.49 / 22.70	61.13 / 60.90	0.34 / 0.30	-0.21 / -0.16
rRNAs	39.23 / 38.61	19.64 / 20.80	16.31 / 16.96	24.82 / 23.63	58.87 / 59.41	0.33 / 0.30	-0.21 / -0.16
*CR1*	27.67 / 26.37	33.17 / 33.43	11.68 / 12.64	27.48 / 27.56	60.84 / 59.80	-0.09 / -0.12	-0.40 / -0.37
*CR2*	27.17 / 26.24	33.20 / 33.00	11.86 / 12.92	27.77 / 27.85	60.38 / 59.23	-0.10 / -0.11	-0.40 / -0.37
CRs	27.42 / 26.30	33.19 / 33.22	11.77 / 12.78	27.62 / 27.71	60.61 / 59.52	-0.10 / -0.12	-0.40 / -0.37

### ﻿Protein-coding genes and codon usage

The lengths of 13 PCGs of *B.kraepelini* and *H.craspedogaster* varied from 159 bp (*atp8*) to 1764 bp (*nad5*) and from 165 bp (*atp8*) to 1782 bp (*nad5*), respectively (Table 2). The A+T content, AT skew and GC skew of the 13 PCGs in *B.kraepelini* and *H.craspedogaster* were 59.01% and 61.92%, 0.203 and 0.122, and -0.463 and -0.415, respectively (Table 3). Excluding terminal codons, a total of 3751 codons were used to encode proteins of *B.kraepelini*, while a total of 3759 codons were used to encode proteins of *H.craspedogaster*. All PCGs started with a standard ATN codon (ATA, ATT or ATG) and ended with the stop codon TAA, AGG, AGA or a single T in both species (Table 2). The incomplete stop codon T was frequently found in both species and in other animal mitogenomes (Ojala et al. 1981; Ki et al. 2010; Tang et al. 2020), which might be the result of post-transcriptional polyadenylation (Donath et al. 2019). Relative synonymous codon usage (RSCU), as a key parameter, was used to evaluate the bias of the synonymous codon, and the values obtained reflecting codon usage preference directly in certain gene samples (Table 4). For *B.kraepelini* and *H.craspedogaster*, the RSCU showed bias toward AT rather than GC at the third codon position. Twenty-five out of all 60 codons were regarded as abundant since these synonymous codons had positive codon usage bias (RSCU value > 1.0). However, the remaining codons, except for the UCU codon (RSCU value = 1.0) in *H.craspedogaster*, had negative codon usage bias (RSCU value < 1.0), and they were considered less abundant codons (Li et al. 2018). Furthermore, *threonine*, *leucine 1*, and *isoleucine* were the most common amino acids, while *cysteine*, *serine 1*, and *aspartic acid* were the least common amino acids in these two species.

**Table 4.** Amino acid composition and relative synonymous codon usage (RSCU) in the mitogenome of *Boigakraepelini* and *Hebiuscraspedogaster*; RSCU values of *B.kraepelini* are shown before the slash (/) and of *H.craspedogaster* are listed after the slash.

Amino acid	Codon	RSCU	Codon	RSCU	Codon	RSCU	Codon	RSCU
Ala (A)	GCC	1.79 / 1.68	GCA	1.59 / 1.56	GCU	0.55 / 0.63	GCG	0.07 / 0.13
Arg (R)	CGA	2.69 / 2.26	CGC	0.63 / 0.52	CGU	0.44 / 0.84	CGG	0.25 / 0.39
Asn (N)	AAC	1.68 / 1.15	AAU	0.32 / 0.85				
Asp (D)	GAC	1.72 / 0.97	GAU	0.28 / 1.03				
Cys (C)	UGC	1.33 / 0.96	UGU	0.67 / 1.04				
Glu (E)	GAA	1.74 / 1.74	GAG	0.26 / 0.26				
Gln (Q)	CAA	1.83 / 1.86	CAG	0.17 / 0.14				
Gly (G)	GGA	1.96 / 1.60	GGC	0.80 / 0.88	GGG	0.73 / 0.70	GGU	0.51 / 0.82
His (H)	CAC	1.66 / 1.41	CAU	0.34 / 0.59				
Ile (I)	AUC	1.22 / 0.90	AUU	0.78 / 1.10				
Leu1 (L1)	CUA	3.16 / 2.25	CUC	0.65 / 0.61	CUU	0.55 / 0.96	CUG	0.41 / 0.23
Leu2 (L2)	UUA	1.09 / 1.68	UUG	0.15 / 0.26				
Lys (K)	AAA	1.89 / 1.79	AAG	0.11 / 0.21				
Met (M)	AUA	1.80 / 1.76	AUG	0.20 / 0.24				
Phe (F)	UUC	1.28 / 1.02	UUU	0.72 / 0.98				
Pro (P)	CCA	2.54 / 2.65	CCC	1.02 / 0.75	CCU	0.28 / 0.44	CCG	0.16 / 0.16
Ser1 (S1)	AGC	0.88 / 0.66	AGU	0.27 / 0.33				
Ser2 (S2)	UCA	2.47 / 2.55	UCC	1.58 / 1.31	UCU	0.63 / 1.00	UCG	0.16 / 0.15
Thr (T)	ACA	1.96 / 1.94	ACC	1.56 / 1.29	ACU	0.41 / 0.65	ACG	0.08 / 0.11
Trp (W)	UGA	1.68 / 1.76	UGG	0.32 / 0.24				
Tyr (Y)	UAC	1.34 / 0.96	UAU	0.66 / 1.04				
Val (V)	GUA	1.70 / 1.71	GUU	0.96 / 1.14	GUC	0.78 / 0.62	GUG	0.56 / 0.52

### ﻿Transfer RNA, ribosomal RNA genes and the A + T-rich region

**Figure 2.**
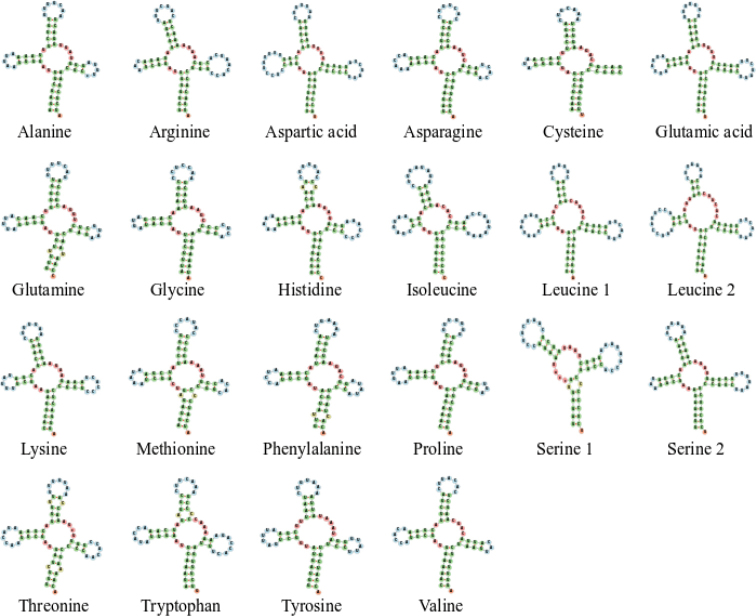
Secondary structure of tRNAs in the mitogenome of *Boigakraepelini*.

**Figure 3.**
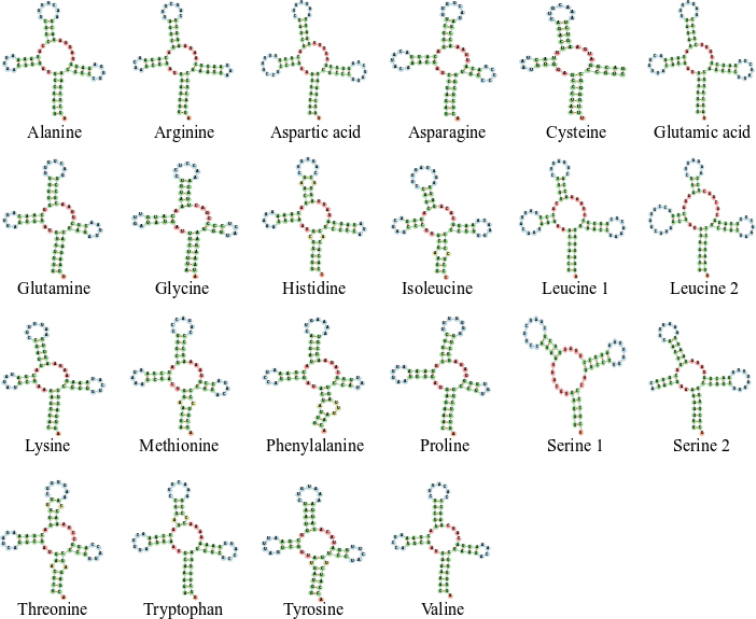
Secondary structure of tRNAs in the mitochondrial genome of *Hebiuscraspedogaster*.

Similar to other snakes, 22 tRNA genes were recovered from the mitogenomes of *B.kraepelini* and *H.craspedogaster*. The tRNA lengths of these two species ranged from 57 bp (*trnS1*) to 73 bp (*trnL2*) (Table 2). The AT content of *B.kraepelini* and *H.craspedogaster* were between 43.94% (*trnI*) and 66.20% (*trnQ*) and 43.75% (*trnI*) and 66.67% (*trnK*), respectively (Suppl. material 1: Table S2). In addition, the tRNA genes of *B.kraepelini* and *H.craspedogaster* had a positive AT skew (0.16 and 0.13, respectively) and a negative GC skew (-0.21 and -0.16, respectively) (Table 3). All tRNA genes, except *trnS1* and *trnC*, showed typical cloverleaf secondary structures (Figs 2, 3). The *trnS1* gene lacked a dihydroxyuridine arm (D arm), and the *trnC* gene lacked the T Ψ C loop. Deletions of the D arm and/or T Ψ C loop in tRNA genes of the mitogenome are known to occur in other Colubridae species (Li 2014). tRNA genes may lack the D arm or the T arm may exhibit lower amounts of peptide production or lower levels of aminoacylation and EF-Tu binding abilities (Watanabe et al. 2014). No pseudogene *trnP* was found between mitochondrial genes *trnI* and *CR2* in either species, although it was present in some snakes (Kumazawa et al. 1998; Dong and Kumazawa 2005; Jiang et al. 2007). Species without pseudogene *trnP* were considered primitive snakes (Wang et al. 2009). Different from the typical arrangement of the mitogenome in vertebrates, here *trnL* (*UUR*) translocated from its original position between *rrnL* and *nad1* to the position between *CR2* and *trnQ*. The rearrangement of the *trnL* (*UUR*) gene is common in Alethinophidia (Dong and Kumazawa 2005; Yan et al. 2008; Chen and Zhao 2009).

As shown in Table 2, the gene *rrnS* in *B.kraepelini* was 917 bp in length and located between *trnF* and *trnV*, while the gene *rrnL* was 1456 bp in length and located between *trnV* and *nad1*. The *rrnS* and *rrnL* genes in *H.craspedogaster* were 8 bp longer and 11 bp shorter, respectively, than those in *B.kraepelini*. These two rRNA genes were AT biased; the A+T content of *rrnS* genes was 55.29% in *B.kraepelini* and 57.08% in *H.craspedogaster*, and the A+T content of *rrnL* genes was 61.13% in *B.kraepelini* and 60.90% in *H.craspedogaster* (Table 3). Both *rrnS* and *rrnL* in the two species showed the same nucleotide composition of the mitogenome: A > C > T > G.

Additionally, similar to some snakes, there were two control regions in both species mitogenomes, in which *CR1* was located between *trnP* and *trnF*, and *CR2* was located between *trnI* and *trnL* (*UUR*). The nucleotide composition and length of the two control regions in the same species were almost identical. The AT skews and GC skews of the two CRs in *B.kraepelini* and *H.craspedogaster* were negative, indicating that T and C were more numerous than A and G (Table 3).

### ﻿Phylogenetic analyses

**Figure 4.**
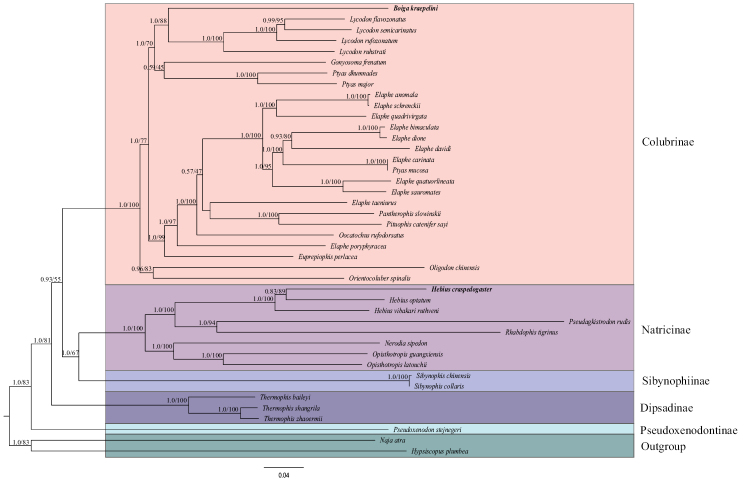
Phylogenetic tree inferred from the nucleotide sequences of 13 mitogenome protein-coding genes using the Bayesian inference (BI) and maximum likelihood (ML) methods. Values on branches separated by slash (/) indicate posterior probability (BI, left) and bootstrap (ML, right).

Phylogenetic trees were constructed based on nucleotide sequences of 13 PCGs in 38 Colubridae species and two outgroups from the families Elapidae and Homalopsidae (Fig. 4). An identical topological structure was produced using both BI and ML methods. Five monophyletic clades that represented five subfamilies, Colubrinae, Natricinae, Sibynophiinae, Dipsadinae and Pseudoxenodontinae, were identified in the family Colubridae. The tree showed a close relationship (BI posterior probabilities [PP] = 1; ML bootstrap [BP] = 67) between Natricinae and Sibynophiinae, and the subfamily Colubrinae was a sister clade of the clade containing Natricinae and Sibynophiinae. These results were consistent with the findings from previous phylogenetic studies (Figueroa et al. 2016; Zaher et al. 2019). In terms of species, *B.kraepelini* was well supported as most closely related to the genus *Lycodon* in the subfamily Colubrinae. In addition, both Figueroa et al. (2016) and Weinell et al. (2021) reported that the genus *Boiga* was the sister group of the genus *Lycodon* based on multiple mitochondrial segments and nuclear genes. *Hebiuscraspedogaster* was clustered with other *Hebius* species and formed a monophyletic clade. The monophyly of the genus *Hebius* was also supported by multilocus (Deepak et al. 2021) and morphological (Hou et al. 2021) phylogenetic analyses.

Both *Boiga* and *Hebius* are species-rich genera in the family Colubridae, with more than 30 species each (Uetz et al. 2022). The phylogenetic relationships within each genus are still unresolved since there are still some species with uncertain systematic positions (Pyron et al. 2013a, 2013b; Deepak et al. 2021). The first mitogenome sequence of *Boiga* and the complete mitochondrial sequence of *H.craspedogaster* from this study will provide more molecular evidence to clarify their taxonomic status and understand potential unknown evolutionary relationships.

## ﻿Conclusions

In this study, we sequenced and characterized the complete mitochondrial genomes of *B.kraepelini* and *H.craspedogaster* for the first time. The mitogenomes of *B.kraepelini* and *H.craspedogaster* were 17,124 bp and 17,120 bp in size, respectively, including 13 PCGs, 22 tRNAs, two rRNAs and two control regions. Both (*B.kraepelini* and *H.craspedogaster*) genome compositions were A+T biased (59.03% and 60.93%, respectively) and showed positive AT skews (0.179 and 0.117, respectively) and negative GC skews (-0.397 and -0.348, respectively). All of the tRNA genes could be folded into typical cloverleaf secondary structures, with the exception of *trnS1*, which lacks the D arm, and *trnC*, which lacks the T Ψ C loop. Phylogenetic analyses were performed with 38 other species from the family Colubridae and two outgroup species. Five clades that represent five subfamilies, Colubrinae, Natricinae, Sibynophiinae, Dipsadinae and Pseudoxenodontinae, were identified. The genus *Boiga* was closely related to the genus *Lycodon*, and both genera belong to the subfamily Colubrinae. *Hebiuscraspedogaster* was clustered with the other two *Hebius* species and closely related to other Natricinae species. This work will be helpful for understanding the evolutionary relationships within the family Colubridae and will provide basic data for the molecular identification of these two species.

## Supplementary materials

Supplementary material 1 Table S1, S2 Data type docx file Explanation note **Table S1.** Primers used for mitogenome amplification of *Boigakraepelini* and *Hebiuscraspedogaster*. **Table S2.** Nucleotide composition of each tRNA of *Boigakraepelini* and *Hebiuscraspedogaster*. https://binary.pensoft.net/file/758214This dataset is made available under the Open Database License (http://opendatacommons.org/licenses/odbl/1.0/). The Open Database License (ODbL) is a license agreement intended to allow users to freely share, modify, and use this Dataset while maintaining this same freedom for others, provided that the original source and author(s) are credited.Shuangshuang Shan, Yu Wang
