# Supplementary material for: ﻿Complete mitochondrial genomes of Boigakraepelini and Hebiuscraspedogaster (Reptilia, Squamata, Colubridae) and their phylogenetic implications
Source: Zookeys. 2022 Oct 17;1124:191–206. doi: 10.3897/zookeys.1124.87861 (PMC9836618; doi:10.3897/zookeys.1124.87861)
Supplement: Supplementary material 1 — Table S1, S2 [file zookeys-1124-191_article-87861__-s001.doc]

**Supplementary material**

**Table S1.** Primers used for mitogenome amplification of *Boiga kraepelini* and *Hebius craspedogaster.*

| No. | Location | Primers | Primer sequences | Size (bp) |
| --- | --- | --- | --- | --- |
| *Boiga kraepelini* | | | | |
| Bk-01 | *CR1* - *rrnS* | Bk-01F | CYGTTCCWAATGGTGTCC | 1573 |
|  |  | Bk-01R | CTGCTAAATCCGCCTTCR |  |
| Bk-02 | *rrnS - trnV* | Bk-02F | TGGGTGGGACAGAATGGGCTAC | 501 |
|  |  | Bk-02R | GGGTGGAGGCTATGATTCTTCT |  |
| Bk-03 | *trnV - nad1* | Bk-03F | TACACTCGAACGACATTA | 2359 |
|  |  | Bk-03R | GTATCGGAATCGTGGGTA |  |
| Bk-04 | *nad1 - nad2* | Bk-04F | CCTAACAGAGGGAGAATCAG | 1753 |
|  |  | Bk-04R | TTGAGGTGTTTATGGCGTTT |  |
| Bk-05 | *nad2 - cox1* | Bk-05F | CACGAGCCACAGAAGCAGCAAC | 1634 |
|  |  | Bk-05R | TGTAATAAAGTTGATTGCTCCC |  |
| Bk-06 | *cox1 - cox2* | Bk-06F | TACGAATAGAACTAACCCAACC | 2298 |
|  |  | Bk-06R | TTCATAGGTGGGTTGTGAGGAT |  |
| Bk-07 | *cox2 - cox3* | Bk-07F | ATCTGCGGAGCAAATCAC | 1746 |
|  |  | Bk-07R | AGGAATATGAACCTCATCAG |  |
| Bk-08 | *cox3 - nad4* | Bk-08F | TTTCGGGTATGAAGCGGCAATC | 1961 |
|  |  | Bk-08R | TTGTGGAATCCTCGGGTAAGAA |  |
| Bk-09 | *nad4 - cob* | Bk-09F | ACCTCACCTGCCTTCAACAAAC | 3852 |
|  |  | Bk-09R | AAGATGGCGACAGATAGGATAA |  |
| Bk-10 | *cob - CR1* | Bk-10F | ACTTCATCCTCCCGTTCATAAT | 1185 |
|  |  | Bk-10R | GTGGGTGTCAGGTGAAAGGTAG |  |
| *Hebius craspedogaster* | | | | |
| Hc-01 | *trnF* - *rrnL* | Hc-01F | AAAGCATAGCACTGAAAATG | 1177 |
|  |  | Hc-01R | AAGGGTTAGTCTTTGCTGTT |  |
| Hc-02 | *trnV* - *nad1* | Hc-02F | TACACCCGAACGACATTA | 2358 |
|  |  | Hc-02R | GTATCGGAATCGGGGGTA |  |
| Hc-03 | *nad1* - *nad2* | Hc-03F | TTCACATCCACCTTAGCA | 2413 |
|  |  | Hc-03R | GTAGTCCCCCCAAGGATAGG |  |
| Hc-04 | *nad2* - *cox1* | Hc-04F | AATGCCCTCAACTCCTCTAA | 2224 |
|  |  | Hc-04R | TTATTCCGCCTACAGTAAAGAG |  |
| Hc-05 | *cox1* - *cox2* | Hc-05F | TYGACAGCCGAGCYTACT | 1473 |
|  |  | Hc-05R | GGCTTCTACTGCGATTGG |  |
| Hc-06 | *cox2* - *cox3* | Hc-06F | GACGACTAAACCAAATCCCA | 1539 |
|  |  | Hc-06R | AGGCATAGGTCGCTTCTTTT |  |
| Hc-07 | *cox3* - *nad4* | Hc-07F | AAATGACCACCAACAGGA | 1901 |
|  |  | Hc-07R | AGTCCGTATCCGCCAAGT |  |
| Hc-08 | *nad4* - *nad5* | Hc-08F | ACCAAAAGCCCACGTAGAAGC | 2022 |
|  |  | Hc-08R | AGTTAGGAATGAGGCGGATA |  |
| Hc-09 | *nad5* - *cob* | Hc-09F | ACCCTAGCTTTCCTACAT | 2347 |
|  |  | Hc-09R | TGGACGGAATGTTATTGATC |  |
| Hc-10 | *cob* - *rrnS* | Hc-10F | ATTTTCAGCACCATTTACCCAC | 1458 |
|  |  | Hc-10R | TGGGAGGTTCAAGACCAAGA |  |

**Table S2.** Nucleotide composition of each tRNA of *Boiga kraepelini* and *Hebius craspedogaster*.

| tRNA | A% | T% | G% | C% | A+T% | AT-skew | GC-skew |
| --- | --- | --- | --- | --- | --- | --- | --- |
| *trnF* | 37.70 / 36.51 | 24.59 / 28.57 | 16.39 / 15.87 | 21.31 / 19.05 | 62.30 / 65.08 | 0.21 / 0.12 | -0.13 / -0.09 |
| *trnV* | 37.50 / 37.50 | 28.13 / 26.56 | 10.94 / 12.50 | 23.44 / 23.44 | 65.63 / 64.06 | 0.14 / 0.17 | -0.36 / -0.30 |
| *trnI* | 27.27 / 26.56 | 16.67 / 17.19 | 27.27 / 26.56 | 28.79 / 29.69 | 43.94 / 43.75 | 0.24 / 0.21 | -0.03 / -0.06 |
| *trnL2* | 39.73 / 41.10 | 21.92 / 21.92 | 15.07 / 15.07 | 23.29 / 21.92 | 61.64 / 63.01 | 0.29 / 0.30 | -0.21 / -0.19 |
| *trnQ* | 39.44 / 38.03 | 26.76 / 21.13 | 9.86 / 12.68 | 23.94 / 28.17 | 66.20 / 59.15 | 0.19 / 0.29 | -0.42 / -0.38 |
| *trnM* | 32.26 / 31.75 | 16.13 / 15.87 | 19.35 / 20.63 | 32.26 / 31.75 | 48.39 / 47.62 | 0.33 / 0.33 | -0.25 / -0.21 |
| *trnW* | 38.46 / 35.82 | 23.08 / 28.36 | 13.85 / 14.93 | 24.62 / 20.90 | 61.54 / 64.18 | 0.25 / 0.12 | -0.28 / -0.17 |
| *trnA* | 33.33 / 27.69 | 26.98 / 29.23 | 14.29 / 16.92 | 25.40 / 26.15 | 60.32 / 56.92 | 0.11 / -0.03 | -0.28 / -0.21 |
| *trnN* | 30.56 / 32.88 | 18.06 / 19.18 | 20.83 / 17.81 | 30.56 / 30.14 | 48.61 / 52.05 | 0.26 / 0.26 | -0.19 / -0.26 |
| *trnC* | 21.67 / 23.73 | 26.67 / 25.42 | 23.33 / 22.03 | 28.33 / 28.81 | 48.33 / 49.15 | -0.10 / -0.03 | -0.10 / -0.13 |
| *trnY* | 35.48 / 33.87 | 30.65 / 29.03 | 12.90 / 17.74 | 20.97 / 19.35 | 66.13 / 62.90 | 0.07 / 0.08 | -0.24 / -0.04 |
| *trnS2* | 31.34 / 31.34 | 28.36 / 28.36 | 14.93 / 14.93 | 25.37 / 25.37 | 59.70 / 59.70 | 0.05 / 0.05 | -0.26 / -0.26 |
| *trnD* | 32.81 / 29.23 | 23.44 / 26.15 | 17.19 / 18.46 | 26.56 / 26.15 | 56.25 / 55.38 | 0.17 / 0.06 | -0.21 / -0.17 |
| *trnK* | 31.25 / 33.33 | 29.69 / 33.33 | 17.19 / 19.05 | 21.88 / 14.29 | 60.94 / 66.67 | 0.03 / 0.00 | -0.12 / 0.14 |
| *trnG* | 34.43 / 32.79 | 26.23 / 31.15 | 16.39 / 14.75 | 22.95 / 21.31 | 60.66 / 63.93 | 0.14 / 0.03 | -0.17 / -0.18 |
| *trnR* | 32.31 / 34.38 | 24.62 / 28.13 | 20.00 / 20.31 | 23.08 / 17.19 | 56.92 / 62.50 | 0.14 / 0.10 | -0.07 / 0.08 |
| *trnH* | 38.46 / 35.38 | 21.54 / 26.15 | 15.38 / 18.46 | 24.62 / 20.00 | 60.00 / 61.54 | 0.28 / 0.15 | -0.23 / -0.04 |
| *trnS1* | 28.07 / 21.05 | 22.81 / 28.07 | 17.54 / 24.56 | 31.58 / 26.32 | 50.88 / 49.12 | 0.10 / -0.14 | -0.29 / -0.03 |
| *trnL1* | 30.99 / 33.80 | 25.35 / 22.54 | 21.13 / 19.72 | 22.54 / 23.94 | 56.34 / 56.34 | 0.10 / 0.20 | -0.03 / -0.10 |
| *trnE* | 33.87 / 36.51 | 24.19 / 25.40 | 17.74 / 14.29 | 24.19 / 23.81 | 58.06 / 61.90 | 0.17 / 0.18 | -0.15 / -0.25 |
| *trnT* | 34.85 / 31.25 | 25.76 / 25.00 | 15.15 / 18.75 | 24.24 / 25.00 | 60.61 / 56.25 | 0.15 / 0.11 | -0.23 / -0.14 |
| *trnP* | 30.65 / 33.87 | 25.81 / 22.58 | 12.90 / 12.90 | 30.65 / 30.65 | 56.45 / 56.45 | 0.09 / 0.20 | -0.41 / -0.41 |

Note: the values for nucleotide composition of *B. kraepelini* are shown before the slash (/) and of *H. craspedogaster* are listed after the slash.
